# Next-Level Prediction of Structural Progression in Knee Osteoarthritis: A Perspective

**DOI:** 10.3390/ijms26104748

**Published:** 2025-05-15

**Authors:** Johanne Martel-Pelletier, Jean-Pierre Pelletier

**Affiliations:** Osteoarthritis Research Unit, University of Montreal Hospital Research Centre (CRCHUM), 900 Saint-Denis, R11.412B, Montreal, QC H2X 0A9, Canada; dr@jppelletier.ca

**Keywords:** osteoarthritis, knee, prognostic, prediction, structural progression, magnetic resonance imaging (MRI), machine and deep learning, biochemical markers osteoarthritis

## Abstract

Osteoarthritis (OA) is a prevalent and disabling chronic disease, with knee OA being the most common form, affecting approximately 73% of individuals over 55 years. Traditional clinical assessments often fail to predict knee structural progression accurately, highlighting the need for improved prognostic methods. This perspective explores the complexity of stratifying knee OA patients based on rapid structural progression. It underscores the importance of such early identification to enable timely and personalized intervention and optimize disease-modifying OA drug clinical trial design, as many trial participants show minimal progression, complicating the assessment of treatment efficacy. We highlight the potential of machine learning (ML) and deep learning (DL) in overcoming this prognostic challenge, as these methodologies enhance classification/stratification capabilities by leveraging multidimensional data and capturing the intricate relationships between diverse features. These include panels of biochemical markers and imaging markers, such as those from magnetic resonance imaging (MRI), as integrating MRI data into ML/DL prognostic models enhances such prediction performance. These automated ML/DL models will offer a transformative approach to stratifying knee OA patients and represent a paradigm shift in disease management. Ultimately, ML/DL applications will not only improve patient outcomes but will also promote innovation in OA research, clinical practice, and therapeutics.

## 1. Introduction

Knee osteoarthritis (OA) is a prevalent and disabling chronic condition characterized by significant pain, reduced mobility, and economic burden. While OA can affect multiple articulations, it predominantly targets weight-bearing joints, with the knee representing the largest clinical proportion—accounting for more than 56% of cases [1]. Although OA can manifest in younger individuals, it primarily affects middle-aged and older adults. The prevalence of knee OA is approximately 22% in individuals around 40 years of age and increases to nearly 73% in those over 55 years [2,3]. OA affects both genders, though women constitute 60% of cases.

The global incidence of OA is rising at an alarming rate. Between 1990 and 2021, cases increased by approximately 137%, with projections suggesting a 114% rise over the next three decades [1]. Currently, OA is the third fastest-growing condition contributing to disability, surpassed only by diabetes and dementia. It is a leading cause of global years lived with disability. It significantly impacts public health systems and individuals by exacerbating comorbidities, including cardiovascular diseases, diabetes, metabolic disorders, and depression, to name a few, likely worsened by reduced mobility and physical inactivity [4,5,6]. These comorbidities, combined with adverse medication effects, contribute to a 55% increase in mortality among OA patients [7,8]. These significantly impact OA management and the treatment of other conditions.

Although the precise etiology of OA remains unclear, several modifiable and non-modifiable risk factors have been identified. Among the non-modifiable factors, age and gender are very important, whereas modifiable factors such as weight significantly influence disease severity. Obesity, in particular, accelerates disease progression and exacerbates symptoms.

**Figure 1:**
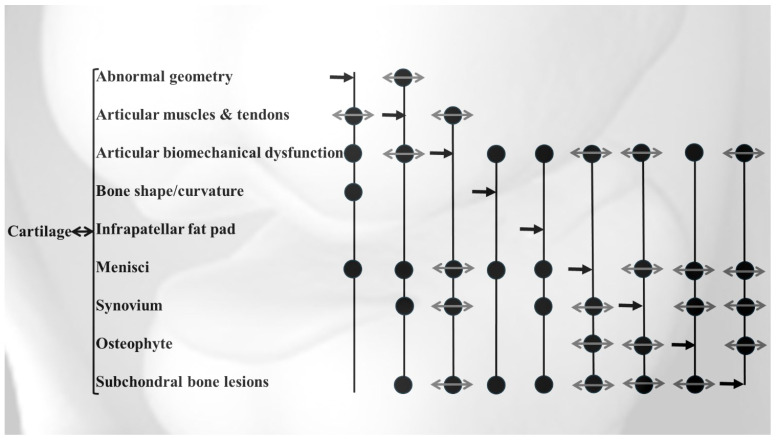
Knee osteoarthritis is a disease affecting the entire joint, involving all major articular tissues, which are interconnected to varying degrees. Notably, the cartilage is associated with all the tissues, while the others are connected to only some. The dot (**●**) marks the specific tissue involved in each relationship, aligning with the corresponding row; the dark double arrow (**↔**) indicates a bidirectional relationship with the tissue in the same row; the gray double arrow (**↔**) represents a bidirectional relationship with the tissue indicated by the straight arrow; and the straight arrow (**→**) signifies a unidirectional relationship between the tissue of interest and those in the corresponding row marked by a dot (**●**).

Radiographically, OA is characterized by joint space narrowing and osteophyte formation at the joint margin. Structurally, the disease was previously shown to primary involve cartilage degradation, subchondral bone alterations, and synovial inflammation with acute flare. However, although these tissue changes are hallmarks of OA, nearly all joint tissues are affected as the disease progresses and are somewhat connected, reinforcing the concept of OA as a disease affecting the entire joint (Figure 1).

Traditionally, OA was described as a slow-progressive condition, often a silent disease, with symptoms starting only after decades of disease evolution. However, recent evidence suggests that disease progression and severity can be rapid in some individuals [9]. Clinical assessments often fail to predict structural progression accurately, and there are no universally accepted criteria for early OA diagnosis and prognosis. Current tools typically identify OA in its moderate stages, when joint structural damage has already occurred, limiting the effectiveness of preventive measures. Existing treatment options primarily focus on symptom management rather than halting disease progression, and no cure or disease-modifying drugs (DMOADs) are currently available.

One of the major challenges in developing DMOADs is the effective selection of trial participants. We lack accurate methods for stratifying patients based on their risk of rapid structural disease progression. As a result, many clinical trial participants recruited already exhibit significant structural damage, and a considerable proportion of patients are in the advanced stages of the disease, showing limited or no disease progression within the trial period. These issues make achieving the statistical power required to evaluate treatment efficacy difficult. Developing tools to identify these patients’ subtypes will allow clinicians to implement targeted prevention strategies and facilitate the inclusion of patients at risk of rapid disease progression in clinical trials. Such a prognosis is therefore critical for both DMOAD trials and early intervention, which has been one of the leading research priorities of some rheumatology organizations and different public–private initiatives [10,11].

## 2. Features for OA Stratification

Given OA’s heterogeneity and multifactorial nature, accurately predicting knee structural progression remains a significant challenge. Clinical phenotyping, which involves careful examination and a detailed understanding of the patient’s natural disease history, provides some insights, but physicians struggle to predict structural disease progression accurately.

Numerous studies have investigated biochemical markers for such stratification of OA patients. However, no single biomarker has proven sufficiently discriminating, neither in the context of diagnosis nor structural disease progression prognosis. Rather than the traditional “one marker—one disease” approach to fully exploit the complex patterns of OA disease, integrating a combination of biomarkers offers a more promising avenue, and recent studies underscore the need for a panel of biomarkers. The usage of circulating biochemical markers derived from biological fluids, such as blood, is particularly valuable due to their accessibility.

Symptoms, such as pain, have also been considered as the outcome for predicting rapid knee structural progression. However, symptom severity does not consistently correlate with structural disease progression, as it often arises later in the disease course when joint damage is already visible on radiographs [12]. Additionally, symptom assessment is inherently subjective.

Other features, such as imaging data, have also been strongly suggested as changes representative of the knee structure. Many models for the outcome of being a structural progressor rely only on radiography measurement. However, radiographic techniques, while commonly employed, have significant limitations. The major ones are this technique’s insensitivity to early knee structure alterations and structure changes over time. Another is the inability to image pathologies in non-bone structures, such as the cartilage, meniscus, and soft tissue, including synovium and infrapatellar fat pad, to name a few [13,14,15].

It is essential to include the evaluation of most knee tissue structures for such an assessment. Although various modalities have been exploited in the evaluation of the knee, magnetic resonance imaging (MRI) emerged as a cornerstone imaging modality for assessing knee OA tissue structures. In addition to revealing the 3D structure of the knee joint, this technology allows precise visualization and quantification of many tissues, providing an unbiased interpretation of OA conditions. Moreover, MRI can detect knee tissue alterations even before radiographic evidence and is highly sensitive to structural change over time [16,17].

## 3. Machine and Deep Learning Approaches in OA Prognosis

Machine learning (ML) and deep learning (DL) offer promising solutions for developing innovative early prognostic models capable of identifying patients at risk of rapid knee structural OA progression. ML refers to a series of mathematical algorithms inspired by the structure and function of the brain that enable the machine to “learn” relationships between input or features and output or outcome data. DL, a subfield of ML, is an advanced technique that uses a layered structure of algorithms to create an artificial neural network that can learn and make intelligent decisions independently. This methodology can extract complex patterns from large datasets. In medicine, ML is mainly used to analyze data from a cohort of patients. DL is also used to analyze data but excels in imaging analysis by automatically extracting relevant image features.

Instead of one-size-fits-all management of the disease, these models will enable a personalized approach by analyzing individual patient data and predicting progression trajectories. In other fields of medicine, ML/DL models have demonstrated significant potential to enhance diagnostic and prognostic outcomes as they can identify features in complex datasets that are important to prediction without requiring assumptions about underlying relationships among features [18,19,20,21,22]. These artificial intelligence methodologies enable pattern recognition beyond human capabilities, outperform traditional logistic regression models, enhance risk stratification, and show robustness in prognosis prediction accuracy.

To develop an “ideal” OA prognostic model that can be integrated into clinical workflows, several key elements must be considered. These include (i) access to harmonized, high-quality datasets; (ii) well-defined ground-truth outcomes—such as incorporating MRI data to reflect knee structural alterations; (iii) comprehensive input variables that combine clinically accessible features (e.g., age, sex, BMI, symptom scores) with known and emerging biomarkers (e.g., for biochemical markers: genomics, epigenomics, transcriptomics, proteomics, and metabolomics, to name a few), which provide insights into underlying molecular changes; (iv) data standardization and model interpretability to ensure usability; and (v) the external validation using independent cohorts to ensure clinical confidence. The integration of these elements is essential for the development of robust ML/DL-based OA prognostic models.

In terms of biomarker inclusion in a model, researchers typically select them based on existing biological knowledge, data availability, or specific hypotheses regarding their potential relevance. ML/DL algorithms then assess and rank these features based on their predictive contribution to the model.

## 4. Machine and Deep Learning OA Prognosis Models That Integrate Biochemical Markers and MRI Data

Approaches to predicting knee OA structural progression have traditionally relied on classical statistical methods. However, this method primarily identifies relationships between features and outcomes without necessarily providing predictive power. In contrast, ML aims to learn patterns directly from data without relying on explicit assumptions. Most of these previous studies largely focus on biochemical markers related to cartilage degradation and synthesis. However, relying solely on cartilage-specific markers does not fully capture the full spectrum of knee tissue alterations that occur during OA. To address this, additional biochemical markers have been incorporated to better reflect changes in knee tissues. For instance, certain interleukins have been used to account for the inflammatory process contributing to joint damage, while metalloproteinases serve as indicators of extracellular matrix breakdown. Some bone markers have also been considered to reflect the subchondral bone changes occurring during disease progression. In these studies, outcomes were mainly based on radiographic evidence, either alone or in combination with clinical data, including pain scores. Only recently have MRI data been integrated as an input feature to capture early structural joint changes or as an outcome measure for OA structural progression analyses.

Recent ML/DL studies focused on predicting knee OA structural progression have investigated biochemical markers beyond the traditional ones as inputs, as well as MRI data alone or in combination with radiographic data as inputs and/or outcomes. The first such study emerged in 2017 (Table 1). As outlined in Table 1, various definitions have been given for the outcome, i.e., individuals at risk of structural progression. Some have employed the WOMAC pain score despite its known limitations. Others have relied on radiography, such as Kellgren–Lawrence grades, joint space narrowing, or width measurements, to apply to subjects with pre-radiographic OA or those in which OA is already present. However, reliance on the semi-quantitative Kellgren–Lawrence system is constrained by practitioner subjectivity. While radiography remains the clinical gold standard for OA assessment, it has notable limitations when used alone to evaluate disease severity, as mentioned above. MRI alone is often used in models by feeding images into a DL algorithm for stratification or outcome prediction, though this requires substantial computational power and expertise. MRI data have also been combined with radiography (joint space narrowing or width) to classify progressors.

Various input features have been utilized in ML/DL models (Table 1) and include MRI-derived imaging markers and biochemical markers (generally with OA risk factors) alone, or combinations of MRI and radiographic data, or MRI, pain score, and biochemical markers. Circulating biochemical markers are valuable inputs for prognostic models, as their fluctuations often precede observable knee structural changes. Numerous biochemical markers reflecting altered joint tissue metabolism have been identified. Notably, in the developed ML/DL models (Table 1), no single marker emerged as the most predictive. Instead, panels of biomarkers were found to be most effective, underscoring the potential of a combined biomarker strategy to improve risk stratification.

As biochemical markers prioritized by the ML/DL algorithms are based on their predictive contribution to the model (Table 1), one model incorporates traditional cartilage-specific markers, namely the serum N-terminal pro-peptide of collagen IIa (a marker of cartilage synthesis) and the urinary C-terminal crosslinked telopeptide type II collagen (a cartilage degradation product) [23]. Another model employs the inflammatory markers C-reactive protein (CRP), monocyte chemoattractant protein-1 (MCP-1), and adipokine leptin [24]. Each of these biomarkers plays a distinct yet interconnected role in OA progression, addressing the multifaceted mechanisms that drive OA progression [25,26,27,28]. CRP, widely recognized as a marker of systemic inflammation, serves not only as an indicator of disease severity but also as a predictor of rapid disease progression [29]. The chemokine MCP-1 plays a critical role in recruiting monocytes and macrophages to inflamed joint tissues and is associated with increased inflammation and degradation of knee tissues [30]. The adipokine leptin, known for its role in energy regulation, has been implicated in OA through its pro-inflammatory effects [31]. It may exacerbate joint inflammation by promoting the synthesis of additional pro-inflammatory mediators.

Genetic biomarkers have also gained interest, with mounting evidence suggesting their involvement in OA progression. As shown in Table 1, one study includes two models, one based on single nucleotide polymorphisms (SNPs) alone, while the other has one SNP along with mitochondrial DNA haplogroups (mtDNA) [32]. A different study employs small non-coding RNAs, specifically microRNAs [33]. Numerous SNPs have been associated with joint integrity and OA progression [34,35,36]. In the study [32], an optimal model identified four SNPs—*TP63*, *DUS4L*, *GDF5*, and *FTO*. These variants contribute to OA pathogenesis through diverse mechanisms. *TP63* variants likely affect the transcriptional regulation of the protein p63, while *DUS4L* variants may influence the post-transcriptional process required for joint stress responses. *GDF5*, one of the most consistently associated SNPs with OA, directly impact cartilage development and repair. Although initially linked to obesity [37], the *FTO* variants may also affect local metabolic processes and inflammatory responses within the joint [38]. Furthermore, the study [32] identified a second model with a similar accuracy that integrates the SNP *SUPT3H* with mtDNA haplogroups. While the OA-related functional role of *SUPT3H* variants remains unclear, mtDNA, which is exclusively maternally inherited, accumulates mutations (haplogroups) over generations, some of which have been linked to either more severe or slower OA progression [39,40,41]. Notably, in the model [32], haplogroup H demonstrated the highest impact, consistent with reports that OA patients with haplogroup H experience a higher incidence and faster disease progression [39,40]. The functional consequences of this haplogroup may be associated with increased free radical production, reduced cell survival under oxidative stress, and increased cell apoptosis, contributing to faster knee tissue degradation [40].

A relatively new class of circulating biomarkers, miRNAs (small non-coding RNA segments of approximately 22 nucleotides), has also shown promise in OA prognosis as being linked to OA severity. miRNAs regulate key processes in joint tissue development, homeostasis, inflammation, cartilage degeneration, autophagy, and apoptosis, among other effects [42,43]. In the developed model [33], four miRNAs were identified, of which only hsa-miR-141-3p and hsa-miR-556-3p were found to be upregulated in OA pathology. Has-miR-141-3p has been associated with increased apoptosis and cartilage destruction [44], although the specific target factors remain uncertain. Has-miR-556-3p is upregulated in the plasma of early-stage OA patients [45]. In OA, it appears to target a protein involved in multiple OA-related signaling pathways [46,47]. Despite no true association with OA being reported for the other two miRNAs and little is currently known about has-miR-3157-5p, hsa-miR-200a-5p was shown to regulate lipid metabolism [48], a process altered in OA.

Some ML/DL models presented in Table 1 rely on a large number of predictive features, many of which are not readily available in routine clinical practice. This complexity can hinder widespread adoption. However, several features, although not currently used in routine practice, could still be assessed through accessible tests. Simplifying these models while maintaining high predictive accuracy is essential for broader clinical implementation. Additionally, many developed models have demonstrated suboptimal performance for clinical application, with only a few achieving strong predictive accuracy. More specifically, Table 1 summarizes 16 studies, some of which developed multiple models using different input or output variables, resulting in a total of 34 models. Among these, only height models demonstrated very good to excellent performance (metric ≥ 0.80). Although not conventional metrics for reporting ML/DL model performance, one model reported a high z-score performance. Another used an s-score, and despite being statistically significant, the predictive value was modest, as reflected by the *p*-value.

Notably, most studies lack external cohort validation (Table 1), which limits model generalization. While many models employ cross-validation, only four have been externally validated. Cross-validation enhances model robustness by testing data on subsets within the same dataset, reduces overfitting risk, and then provides a more reliable estimate of the model’s performance. However, since it relies on the same dataset, it does not fully capture real-world variations. In contrast, external validation using an independent dataset, which could be from the same cohort or different healthcare settings, time periods, or populations, provides a true test of generalization. It ensures that the model is evaluated on entirely unseen data, which better reflects its real-world applicability. A desirable approach involves a two-step validation process: cross-validation for model selection and tuning, followed by external validation to assess final model performance and generalizability. However, while external validation is important, its effectiveness may be limited by the fact that some cohorts may not represent the broader population. For instance, the prevalence of knee OA risk factors in a cohort such as the Osteoarthritis Initiative (OAI) (the most used to date) is higher than in community-based cohorts [49,50]. Consequently, models that perform well in high-risk populations may not generalize to lower-risk or more diverse patient groups. Similarly, a model that performs well on a community-based population may not be as effective for individuals at high risk of knee OA. Ideally, comparing model performance across multiple external validation cohorts would enhance clinical applicability, improve generalizations, and facilitate integration into clinical practice.

The limitation of not using an external cohort in the OA ML/DL models that incorporate MRI data likely stems from the restricted availability of MRI data, which is often confined to specific OA cohorts. This also explains why the OAI cohort is the most commonly used dataset for OA modeling, in addition to being highly comprehensive. OAI is a multicenter, ten-year observational study of 4796 men and women sponsored by the National Institutes of Health [49]. It provides an extensive dataset documenting the natural history of knee OA of at-risk subjects, those with early or preclinical disease, and patients with established OA. The dataset includes a wide range of demographic and clinical data, patient-reported outcomes, imaging (X-rays and MRI acquired during this study), biochemical and genetic markers, and various risk factors. Additionally, it contains information on other variables, including pharmacological treatments, food questionnaires, physical examinations, gait parameters, quadriceps strength, smoking status, educational level, yearly income, and many others.

While there is currently no universal consensus on a specific performance threshold that defines a “clinically acceptable” prognostic model for knee OA, it is recommended that achieving an AUC/accuracy ≥ 0.80, particularly when paired with strong external validation on independent cohorts, may serve as an initial benchmark. When combined with model transparency, generalizability, and ease of clinical integration, such a performance level is increasingly considered necessary to support adoption by the broader clinical and research communities.

**Table 1:** Models for knee osteoarthritis structural progressor prediction using magnetic resonance imaging and machine/deep learning.

Author/Year	Purpose of the Study	Cohort	Learning Algorithm for the Final Model	Best Predictive Input Features	Outcome (Progressors) Definition	Number of Participants	Best Prediction Performance for the Progressors	Validation with an External Cohort
Hafezi-Nejad et al./2017 [51]	To investigate the association between baseline lateral femoral cartilage volume and medial joint space loss progression	FNIH (subset of OAI)	Multi-layer-Perceptron (MLP)	24–48-month changes in the lateral femoral plate cartilage volume	Medial joint space loss ˃ 0.7 mm progression 1. Baseline 2. 24-month change	Progressor: 297 Non-progressor: 303	**AUC** 1. 0.63 2. 0.67	No
Du et al./2018 [52]	To explore the hidden biomedical information from knee MRI for OA progression prediction	OAI	1. Principal Component, Artificial Neural Network (ANN), Support Vector Machine (SVM), Random Forest (RF), Naïve Bayes (NB)	Features from 18 medial compartments showed better performances than the 18 lateral features. The total 36 features generated the best performance	Change over two years of: 1. Kellgren–Lawrence grade 2. JSN grade on medial compartment 3. JSN on lateral compartment grade	Progressor: 100 Non-progressor: 100	**AUC** 1. 0.76 2. 0.79 3. 0.70	No
MacKay et al./2018 [53]	To assess if a change in MRI subchondral bone texture is predictive of radiographic knee OA progression	OAI	Subchondral bone texture using radiomic approach	12–18-month follow-up change in subchondral bone texture features when tibial and femoral data are combined	Decrease minimal JSW ≥0.7 mm 1. At 36 months (initial change) 2. Change between 36 and 72 months	**Baseline** Progressor: 61 Non-progressor: 61 **12–18-month follow-up** Progressor: 53 Non-progressor: 52	**AUC** 1. 0.65 2. 0.68	No
Nelson et al./2019 [23]	To define the progression of OA phenotypes potentially more responsive to interventions	FNIH (subset of OAI)	Distance Weighted Discrimination (DWD), Direction-Projection-Permutation (DPP), Clustering Methods	Baseline variables with the most significant contribution 1. To non-progression: WOMAC pain score, lateral meniscal extrusion, and serum N-terminal pro-peptide of collagen IIA 2. To progression: bone marrow lesions, osteophytes, medial meniscal extrusion, and urine C-terminal crosslinked telopeptide type II collagen	Medial JSN ≥0.7 mm and WOMAC total score increase ≥9 points at 48 months	Progressor: 192 Non-progressor: 200	**Z Score**: 10.1	No
Jamshidi et al./2020 [54]	To identify the most important features of structural knee OA progressors	OAI	Six machine learning: Least Absolute Shrinkage and Selection Operator (LASSO), Elastic Net Regularization (ENR), Gradient Boosting Machine (GRM), Random Forest (RF), Information Gain (IF), Multi-Layer Perceptron (MLP)	Baseline medial minimum JSW, MRI-based mean cartilage thickness of peripheral, medial and central tibial plateau, and medial JSN as a score	1. JSN ≥1 mm at 48 months 2. Medial plateau cartilage volume loss as evaluated by MRI at 96 months 3. Medial plateau cartilage volume loss as evaluated by MRI at 48 months 4. Kellgren-Laurence grade ≥2	1. Progressor: 620 Non-progressor: 200 2. Progressor: 795 Non-progressor: 803 3. Progressor: 514 Non-progressor: 530 4. Progressor: 811 Non-progressor: 657	**AUC** 1. 0.92 2. 0.73 3. 0.70 4. 0.87	No
Bonakdari et al./2021 [24]	To build a comprehensive gender-based machine learning model for early prediction of at-risk knee OA patient structural progressors using baseline serum levels of adipokines/related inflammatory factors and age and BMI	OAI	Support Vector Machine (SVM)	Age, BMI, C-reactive protein/monocyte chemoattractant protein-1 and leptin/C-reactive protein	Prediction of knee OA structural progressors as in [54], in which the inputs were baseline medial minimum JSW, MRI-based mean cartilage thickness of peripheral, medial and central tibial plateau, and medial JSN as a score	Progressor: 357 Non-progressor: 320	**Accuracy** ≥ 0.81	Cohort: Naproxen arm of the Licofelone clinical trial [55] Progressor: 30 Non-progressor: 14 **Accuracy**: ≥ 0.92
Schiratti et al./2021 [56]	To develop a proof-of-concept predictive model for OA radiographic progression and knee pain	OAI	Multi-Layer-Perceptron (MLP)	1. Medial joint space 2. Intraarticular space where effusion is observed	1. OA progression defined as minimum JSN loss of at least 0.5 mm at 12 months 2. Pain prediction (WOMAC pain score ≥2 points)	Knees: 9280	**AUC** 1. 0.63 2. 0.72	No
Bonakdari et al./2022 [57]	To assess if baseline knee bone curvature could predict cartilage volume loss at one year. Development of a gender-based model	OAI	Adaptive Neuro-Fuzzy Inference System (ANFIS)	Baseline bone curvature regions of the lateral tibial plateau, medial central condyle, lateral posterior condyle, and lateral and medial trochlea	Twelve global or regional knee cartilage volume losses at one year (global knee, femur, condyle, tibial plateau; lateral compartment, femur, condyle, tibial plateau; medial compartment, femur condyle and tibial plateau)	Progressor as defined in [54], in which the inputs were baseline medial minimum JSW, MRI-based mean cartilage thickness of peripheral, medial and central tibial plateau, and medial JSN as a score	**Accuracy** 0.92–0.79	Cohort: Naproxen arm of the Licofelone clinical trial [55] Progressor: 53 **Accuracy:** 0.96–0.79 except for medial tibial plateau for women
Bonakdari et al./2022 [32]	To evaluate if single nucleotide polymorphism genes and mitochondrial DNA haplogroups/clusters could predict early knee osteo-arthritis structural progressors	OAI	Support Vector Machine (SVM)	1. Age, BMI, *TP63, DUS4L, GDF5, FTO* 2. Age, BMI, mitochondrial DNA haplogroup (H, J, T, Uk, and others), *FTO*, *SUPT3H*	Prediction of knee OA structural progressors as in [54], in which the inputs were baseline medial minimum JSW, MRI-based mean cartilage thickness of peripheral, medial and central tibial plateau, and medial JSN as a score	Progressor: 276 Non-progressor: 625	**Accuracy** 1. 0.85 2. 0.83	Cohort: TASOAC [58] Progressor: 71 Non-progressor: 158 **Accuracy** 1. 0.81 2. 0.86
Hu et al./2022 [59]	By using an adversarial evolving neural network to estimate longitudinal knee OA prediction	OAI	Adversarial evolving neural network (A-ENN)	Evolving traces of Kellgren–Lawrence grades determined with a discriminator for longitudinal grading	An increase >1 in the Kellgren–Lawrence grade compared to baseline	Knees: 3294	**Accuracy** Overall: 0.63 Baseline: 0.65 12 months: 0.65 24 months: 0.64 36 months: 0.62 48 months: 0.60	No
Panfilov et al./2022 [60]	To predict knee OA progression from structural MRI using deep learning	OAI	Convolutional Neural Network (CNN)-Transformer	Aggregation of features	Changes in Kellgren–Lawrence grade within 96 months with three classes: No progression within 96 months Slow progression (after 72 and within 96 months) Fast progression within 72 months	Knees: 4866	**AUC** 0.78	No
Costello et al./2023 [61]	To develop a machine learning model incorporating gait and physical activity to predictmedial tibiofemoral cartilage worsening over 2 years	MOST	An ensemble machine learning approach using: Bayesian Adaptive Regression Trees (BART), Generalized Linear Model (GLM), Least Absolute Shrinkage and Selection Operator (LASS0) Ridge Regression (RR), Elastic Net (E-Net), Random Forest (RF), and Extreme Gradient Boosting (XGBoost)	High lateral ground reaction force impulse, more time spent lying and low vertical ground reaction force unloading rate	Cartilage worsening over 2 years: area and/or dept in at least one of the five medial tibiofemoral subregions	Participants: 947 with Kellgren-Laurence ≤2, 133 experienced cartilage worsening over 2 years	**AUC** 0.73	No
Hu et al./2023 [62]	To develop a deep-learning method for predicting the progression of knee OA based on MR images	FNIH (subset of OAI)	DenseNet169	Patellofemoral joints, meniscus, infrapatellar fat pad, muscles posterior	Loss in medial minimum joint space width ≥0.7 mm from baseline to 24, 36, 48 months and pain progression: WOMAC pain subscale defined as a persistent increase from baseline to 24, 36, 48 months of ≥9 points on 1–100 score	Progressor: 182 Non-progressor 182	**AUC** Baseline 0.66 12 months: 0.74 24 months: 0.78	No
Jansen et al./2023 [63]	To predict 2-year structural progression	IMI-APPROACH (participants from five observational cohorts, namely, CHECK, HOSTAS, MUST, PROCOAC, and DIGICOD)	Random Forest (RF)	Minimum JSW decrease > 0.3 mm/year	Different parameters were used: Minimum JSW decrease > 0.3 mm/year, MRI data used the MOAKS scores (0–3) of the five medial or lateral tibiofemoral subregions were summarized to one score for each feature and included only if all subregions in the compartment could be scored. Progression was defined as an increase of at least one full score in the most affected compartment (MAC)	Participants: 237 Participants were progressors if at least one of two areas in the most affected compartment surpassed the progression cut-off (JSW predefined threshold). Accordingly, the number of progressors was 14–86, according to the cohort	**s-score for progressor**: It significantly predicts minimum JSW progression (*p* ≤ 0.03). It could not predict structural progression based on the predefined criterion or the smallest detectable change	No
Jamshidi et al./2024 [33]	To develop a miRNA prognosis model for identifying knee OA structural progressors using integrated machine/deep learning tools	OAI	Artificial Neural Network (ANN)	Age, has-miR-141-3p, has-miR-556-3p, has-miR-200a-5p, has-miR-3157-5p	Prediction of knee OA structural progressors as in [54], in which the inputs were baseline medial minimum JSW, MRI-based mean cartilage thickness of peripheral, medial and central tibial plateau, and medial JSN as a score	Progressor (*n* = 91) Non-progressor (*n* = 61)	**AUC**: 0.94 **Accuracy**: 0.84	Independent OAI cohort Progressor (*n* = 14) Non-progressor (*n* = 16) **AUC**: 0.81 **Accuracy**: 0.83
Lv et al./2025 [64]	Using a longitudinal MRI on 32 sub-structural texture-guided graph convolution networks to improve progression prediction of knee OA	FNIH (subset of OAI)	1. Longitudinal MRI sub-structural texture-guided graph convolution network (LMSST-GCN) with clinical data (ceLMSST-GCN) 2. Support Vector Machine (SVM) 3. Random Forest (RF) 4. Extreme Gradient Boosting (XGBoost)	Loss of cartilage and sclerosis of subchondral bone at the tibial medial central region, the injury of lateral meniscus, and abnormal changes in the infrapatellar fat pad	Radiographic progression: combination of radiographic progression (JSW ≥0.7 mm) and WOMAC pain progression (increase of at least 9 points [0–100 scale] at 2 or more timepoints from baseline to 24, 36, 48 months) at 4-year follow-up compared to baseline (pain progression occurred at the third and fourth year of follow-up	Progressor: 194 Non-progressor: 406	**AUC (multi-timepoints)** 1. 0.82 2. 0.73 3. 0.74 4. 0.75	No

Modified and updated from Table A2.B: Martel-Pelletier et al., Ther Adv Musculoskelet Dis 2023 [65], under a CC-BY-NC Creative Commons License. BMI, Body mass index; CHECK, Cohort Hip and Cohort Knee; DenseNET, Dense convolutional network; DIGICOD, Digital Cohort Osteoarthritis Design; DUS4L, Dihydrouridine synthase 4-like; FNIH, Foundation for the National Institutes of Health OA Biomarkers Consortium; FTO, FTO alpha-ketoglutarate-dependent dioxygenase; HOSTAS, Dutch Hand OsteoArthritis in Secondary care; GDF5, Growth differentiation factor; IMI-APPROACH: Innovative Medicine’s Initiative Applied Public–Private Research enabling OsteoArthritis Clinical Headway; JSN, Joint space narrowing; JSW, Joint space width; OA, Osteoarthritis; Licofelone, Drug that inhibits the cyclooxygenase and 5-lipoxygenase pathways; MUST, Musculoskeletal pain in Ullensaker STudy; OAI, Osteoarthritis Initiative; miRNA, microRNA; mm, millimeter; MRI, Magnetic resonance imaging; MOST, Multicenter Osteoarthritis Study; PROCOAC, PROspective COhort of A Coruña; SUPT3H, SPT3 homolog; TP63, Tumor protein P63; WOMAC, Western Ontario and McMaster universities osteoarthritis index.

## 5. Limitations of OA Prognosis Machine and Deep Learning Models

While some ML/DL models demonstrate high performance in predicting knee OA progression, reproducibility and clinical applicability remain key challenges. Another limitation is the reliance on the standard approach of visually interpreting X-ray imaging to assess joint space narrowing or width and to perform the Kellgren–Lawrence grading. These methods are prone to inter-clinician variability, leading to inconsistent assessments [66,67]. Automating these readings will improve data reliability and, consequently, model performance. Studies have demonstrated the feasibility of such automation using, for example, DL and radiomics techniques [68,69,70,71,72]. Although these approaches enhance objectivity, accuracy, and reproducibility, they are not yet standard practice.

MRI variability in MRI acquisition protocols, segmentation techniques, and data collection processes also poses challenges. Moreover, MRI is not routinely used in all clinical venues for knee OA evaluation, limiting the applicability of models that depend on MR-based features. Similarly, biochemical marker collection and assay inconsistencies further hinder model reproducibility and external applicability. Establishing standardized protocols is crucial to reducing discrepancies and enhancing the consistency and external reproducibility of the models and, therefore, the model applicability. Moreover, more comprehensive usage of biochemical markers, including those that are not necessarily intuitive or are emerging, will help identify new patterns associated with knee OA progression in addition to OA research.

Another limitation is the lack of public access to the source code and model parameters. Greater transparency and data-sharing initiatives would allow independent validation. Although the dataset from the OAI is publicly available, only three models in Table 1 indicate data availability in a repository [33,56,61], and two provided open-source code [60,64]. Encouraging researchers to develop and share ready-made software will accelerate clinical adoption.

## 6. Path Toward Clinical Translation of Machine and Deep Learning Models

To contextualize the path toward clinical translation of ML/DL prognostic models in OA, the following outlines key considerations for their adoption in clinical practice.

The successful implementation of predictive ML/DL models for OA prognosis depends on clinician trust, interpretability, usability, and evidence of clinical value [73,74]. Clinicians are becoming increasingly receptive to artificial intelligence-driven tools, primarily when these models address current limitations, for example, in patient stratification for prognostic evaluation in OA. For clinical acceptance, models must provide transparent outputs, improve patient stratification, and support decision-making.

Despite this growing openness, several hurdles remain for clinical adoption. Predictive ML/DL models used in healthcare are now recognized as medical devices and are subject to regulatory oversight under frameworks such as the U.S. Food and Drug Administration, Health Canada, and European Medical Device Regulation [75,76,77,78]. To gain regulatory approval, these models must demonstrate not only high predictive performance but also safety, reproducibility, and generalizability across diverse populations and clinical settings. In addition, high-performing ML models must undergo rigorous clinical validation, ideally through prospective studies or randomized controlled trials. As discussed earlier, external validation on independent cohorts is critical for ensuring that the model generalizes beyond the original training cohort. Another major challenge is integration into clinical workflows, particularly within Electronic Health Record systems. Usability, intuitive interface design, and interoperability with existing hospital infrastructure are essential for successful implementation. Furthermore, ethical deployment of ML/DL tools must address data security, informed consent, and the mitigation of algorithmic bias to ensure equitable patient care.

Currently, in the musculoskeletal field, to our knowledge, ML tools for prognosis have not yet achieved regulatory adoption, unlike in other medical domains (e.g., Sepsis Watch, for predicting sepsis risk in hospitalized patients [79]). However, in musculoskeletal radiology, although ML tools diagnostic applications are currently more common and further along in terms of regulatory approval, prognostic tools are still emerging and under evaluation or being piloted as decision-support modules. These developments highlight the growing momentum toward clinical adoption in this field.

## 7. Conclusions

Aligning patients with optimal and personalized interventions tailored to their individual profiles remains a critical goal in OA management. ML/DL-based models integrating different features, particularly biomarkers and MRI data, represent a paradigm shift in disease prognosis and clinical decision-making. Bridging molecular insights with structural changes has increased the translation of research to patients and will facilitate early OA patient stratification. Moreover, a multifaceted approach to biochemical markers, encompassing proteins and genetic and molecular indicators, is necessary to capture the complex pathophysiology of OA and develop accurate and clinically applicable models.

Developing the accessibility of automated models will provide healthcare professionals with early warning systems for identifying patients at risk of rapid OA progression. Such prognosis models also have the potential to optimize DMOAD clinical trial designs by reducing costs, shortening timelines, and providing faster results. Consequently, they could accelerate advancements in OA therapeutics by broadening treatment evaluation opportunities.

Overcoming the current challenges, as discussed above, will be essential for translating these technologies into real-world practice, the implementation of which is essential to fully harness the potential of knee OA structural progressor prognosis. However, progress in this field will require sustained collaboration between scientists, bioinformaticians, and clinical experts. Proactively fostering such interdisciplinary efforts will help mitigate the impact of OA, enhance patient outcomes, and improve their quality of life.

In summary, prioritizing the development, validation, and open-access application and dissemination of prognostic tools for identifying patients at risk of rapid structural progression in OA will be instrumental in driving innovation across research and clinical domains. These tools have the potential to significantly enhance patient care, inform clinical management strategies, support the design of targeted clinical trials, and accelerate the development of novel therapies; all of these will contribute to acting sooner than the disease.

## Data Availability

Not applicable.
